# Histidine–tryptophan–ketoglutarate solution versus multidose cardioplegia for myocardial protection in cardiac surgeries: a systematic review and meta-analysis

**DOI:** 10.1186/s13019-022-01891-x

**Published:** 2022-05-31

**Authors:** Muayad Albadrani

**Affiliations:** Department of Family and Community Medicine, College of Medicine, Taibah University, Al-Madinah Al-Munawwarah, Kingdom of Saudi Arabia

**Keywords:** HTK solution, Custodiol solution, Cardioplegia, Cardiac surgery, Meta-analysis

## Abstract

**Background:**

Surgical procedures in the heart requires protection of the heart from ischemia–reperfusion injury. Cardioplegia is the primary myocardial protective method in use. Histidine–tryptophan–ketoglutarate (HTK) solution is an intracellular cardioplegic solution that was initially used to preserve organs for transplantation.

**Methods:**

A systematic electronic search was conducted in July 2021, in four databases; PubMed, Scopus, Web of Science, and Cochrane Library for eligible randomized controlled trials. The results were screened and the eligible trials were identified. Thereafter, the relevant data were extracted and pooled as mean difference or risk ratio, and 95% confidence interval in an inverse variance method using RevMan software.

**Results:**

This review included 12 trials (n = 1327). HTK solution has resulted significantly in shorter intensive care unit stay (MD = − 0.09; 95% CI [− 0.15, − 0.03], p = 0.006), and shorter hospital stay (MD = − 0.51; 95% CI [− 0.71, − 0.31], p < 0.00001). Moreover, the patients who received the HTK solution had significantly lower levels of creatine kinase (after 4–7 h (MD = − 157.52; 95% CI [− 272.31, − 42.19], p = 0.007), and 24 h (MD = − 136.62; 95% CI [− 267.20, − 6.05], p = 0.04)), as well as creatine kinase muscle brain band (after 44–48 h (MD = − 3.35; 95% CI [− 5.69, − 1.02], p = 0.005)).

**Conclusion:**

HTK solution had the same efficacy and safety as other cardioplegic solutions in most of the clinical parameters. Furthermore, the solution showed superiority in fastening the recovery and protecting the myocardium at the biochemical level. HTK solution provides longer myocardial protection; therefore, it limits surgical interruption. HTK solution can be used as an alternative to the currently used cardioplegic solutions.

## Introduction

Myocardial damage is a major concern that accompanies cardiac surgeries. This damage is often multifactorial, but among the main attributing factors is the ischemia–reperfusion injury [1]. Myocardial damage can result in arrhythmias, myocardial infarction, or low cardiac output syndrome. As a result, major cardiac and renal morbidities, extended intensive care unit (ICU), and hospital stays, and a higher risk of mortality might occur [1, 2]. To avoid these consequences, several myocardial protective methods were introduced, with the aim of minimalizing ischemia–reperfusion injury. Cardiac protection methods work by decreasing the cardiac metabolic demand, which improves its tolerance to ischemia. The main methods are chemical arrest (cardioplegia), topical hypothermia, and limiting myocardial edema.

Cardioplegia is the primary cardiac protection method in use. It is applied through the injection of a cardioplegic solution that causes heart diastolic arrest. Cardioplegic solutions can be divided into two types based on their molecular composition. Extracellular solutions, which include high amounts of sodium, calcium, potassium, magnesium, and bicarbonate, are the first type. These arrest the heart by depolarizing the myocardial membrane. In the second category, there are the intracellular solutions, sodium and calcium levels are low in this type. These induce a hyperpolarizing arrest of the myocardium [3, 4].

Histidine–tryptophan–ketoglutarate (HTK) solution (Custodiol/Bretschneider) is an intracellular cardioplegic solution, introduced in the 1970s. The HTK solution was initially used to preserve organs for transplantation, thereafter, it was used in cardioplegia [5–7]. Added to its hyperpolarizing arrest that mimics the normal cardiac resting, histidine, tryptophan, ketoglutarate, and mannitol are all present in this solution. Each of these components adds an extra value in protecting the myocardium. Histidine buffers the ischemia-induced acidosis, therefore, improves the anaerobic glycolysis. Tryptophan is an effective cell membrane stabilizer. Ketoglutarate is a Krebs cycle intermediate, which enhances energy production and recovery following reperfusion. Moreover, mannitol minimizes cellular edema by maintaining the cellular environment osmolality, in addition to being a free radical scavenger [8, 9].

A single dose of the HTK solution provides over two hours of myocardial protection. This feature allows time-saving and avoidance of surgical interruption for re-administration of the solution (as in other cardioplegic solutions, which protects for only 20 to 30 min) [10]. However, the use of the HTK solution in cardioplegia is still an off-label indication in many countries. Therefore, this systematic review and meta-analysis aims to provide updated evidence of the efficacy and safety of HTK solution in comparison with other alternative solutions in cardiac surgeries.

## Methods

This systematic review and meta-analysis was performed in accordance with Cochrane Handbook for Systematic Reviews of Interventions [11]. Thereafter, the report was written following the preferred reporting items for systematic reviews and meta-analyses (PRISMA) statement [12].

### Literature search

A systematic search was conducted in four electronic databases: Medline via PubMed, Scopus, Web of Science, and Cochrane Central Register of Controlled Trials. The databases were searched from their inception through November 2021, using the following terms: (histidine–tryptophan–ketoglutarate solution; HTK solution; HTK solution of Bretschneide; Bretschneider solution; Custodiol solution) AND (Crystalloid Cardioplegia; Blood Cardioplegia) AND (Heart Surgical Procedures; Procedure, Cardiac Surgical; Procedures, Cardiac Surgical; Surgical Procedure, Cardiac; Surgical Procedures, Cardiac; Surgical Procedures, Cardiac; Surgical Procedures, Cardiac; Surgical Procedures, Cardiac; Surgical Procedures, Cardiac; Surgical Procedures, Cardiac; Surgical Procedures, Cardiac; Surgical Procedures, Cardi*).

### Eligibility criteria and studies selection

This review included the randomized controlled trials (RCTs) that compared HTK solution to another cardioplegic solution in any cardiac surgery. Conference abstracts, thesis, and non-English studies were excluded from this review.

The duplicates were deleted from the search results, and a double-step screening was performed. Initially, the titles and abstracts of the retrieved articles were screened. Full-text screening was then performed for final eligibility.

### Quality assessment (risk of bias)

The included trials were assessed for potential risk of bias using the Cochrane tool of Cochrane handbook for systematic reviews of interventions [13]. This tool assesses the risk of bias in six domains: (1) Random sequence generation (selection bias); (2) Allocation concealment (selection bias); (3) Blinding of participants (performance bias); (4) Blinding of assessors (detection bias); (5) Incomplete data (attrition bias); (6) selective reporting (reporting bias), in addition to any other potential source bias.

### Data extraction

A summary of the trials key features, baseline data of the enrolled patients, and the treatment outcomes of efficacy and safety were extracted from the included trials. The assessed outcome included: cardiopulmonary bypass (CBP) time, aortic cross-clamping time, cardiac arrest beginning time, number of grafts, postoperative inotropic support, ejection fraction (EF) change, electrocardiogram (ECG) changes, postsurgical atrial fibrillation, hospital and ICU stay, in addition to creatine kinase (CK), creatine kinase muscle brain band (CK-MB), and troponin-I (Tn-I) levels.

### Data synthesis and analysis

The statistical analysis of this review was conducted using the RevMan software (version 5.2; Cochrane Collaboration, Oxford, UK). Continuous data were pooled as mean difference (MD) and 95% confidence interval (CI), whereas dichotomous data were pooled as risk ratio (RR). Heterogeneity among the included trials was evaluated by visually inspecting the forest plot. Additionally, the I-squared (I^2^) and chi-squared statistics were used. An I^2^ value of ≥ 50% indicates statistical heterogeneity, in this case, a random-effect model is used instead of the fixed-effect model [14, 15].

## Results

### Literature search and characteristics of the included trials

**Fig. 1:**
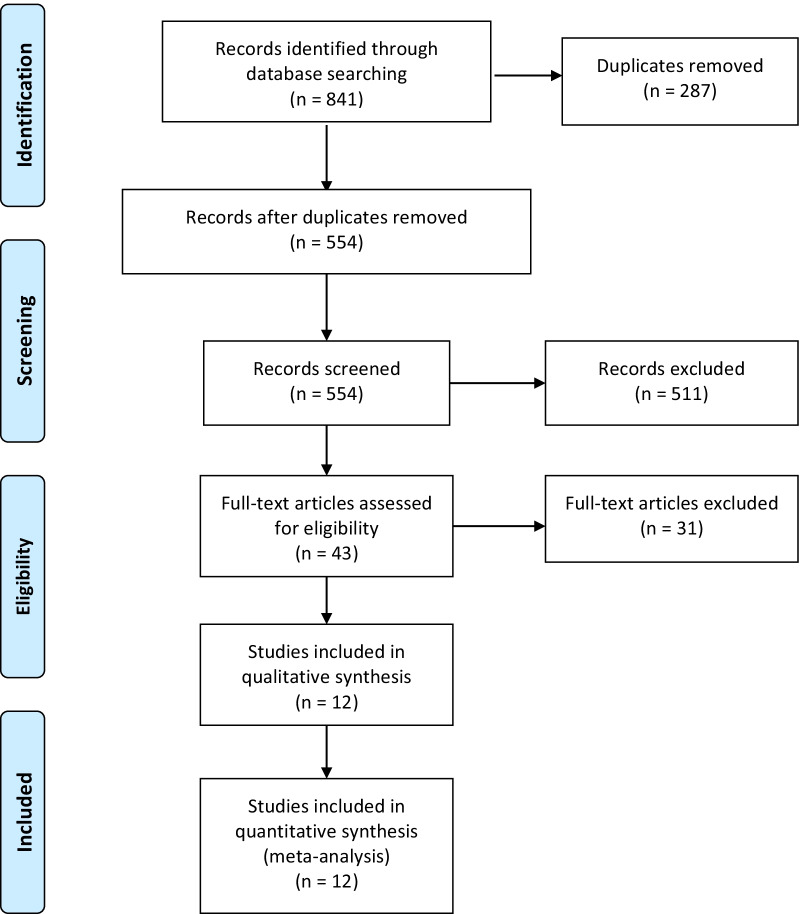
PRISMA flow diagram

Our systematic electronic databases search retrieved 841 articles. After removing the duplicates, 554 articles were screened. By title and abstract screening, 511 articles were excluded. Another 31 articles were excluded by full-text screening. Finally, 12 trials [6, 10, 16–25] were included in our qualitative and quantitative synthesis (Fig. 1). A total of 1,327 patients were enrolled, among them, 666 patients had received the HTK solution.

The included trials compared HTK solution to other cardioplegic solutions—which require multiple doses (multiple doses cardioplegia (MDC))—in various cardiac surgeries. A summary of the included trial key feature, and baseline characteristics of the enrolled patients are presented in Tables 1, and 2 respectively.

**Table 1:** Shows summary of the included trials key features

Study ID	Country	Sample size	Eligibility criteria	HTK solution administration	Type of MDC	MDC administration
Ali et al. [16]	Egypt	320 patients (160 in each group)	The study included patients aged between 30 and 70 years, who were posted for various cardiac surgeries. Patients having unstable angina (class III/IV), LVEF < 40%, acute MI, renal failure history, or emergency cardiac surgery were excluded	30 ml/kg of the solution were administered over 6–7 min, at 4 °C, through an antegrade route. The solution was delivered within 12–15 min at an initial pressure of 80–100 mmHg, which was reduced to 40–60 mmHg after myocardial arrest	Blood	One liter of the 4:1 (blood:crystalloid) mixture was administered at ≤ 29 °C. The mixture was delivered through an antegrade route at a pressure of 80–100 mmHg, and was repeated every 30–45 min. Before myocardial perfusion, another warm blood retrograde dose was administered
Cvetković et al. [17]	Serbia	104 patients (54 for HTK, and 50 for MDC)	The study included adult patients undergoing CABG, having at least two angiographic graftable target vessels (> 2.0 mm in diameter, with ≥ 70% stenosis), LVEF ≥ 30%, and normal valves. Patients > 80 years, having MI within a month of the operation, reoperation, medical emergency off-pump CABG, ongoing myocardial ischemia, pericarditis, coronary endarterectomy, LV surgical restoration, left main stenosis > 50%, or serum creatinine > 200 µmol/L were excluded	20 mL/kg of the solution were administered over 6–8 min, at 4–8 °C, through an antegrade route	Crystalloid	An initial one liter of St.Thomas solution was administered, over 3–5 min, at 4–8 °C, through an antegrade route. This was followed by maintenance doses of 200 mL over 2 min every 20 min
Vivacqua et al. [18]	US	110 patients (55 in each group)	The study included patients undergoing a cardiovascular surgery that needs cardioplegia. Previous cardiovascular surgery, pregnancy, medical emergency, and dialysis were exclusion criteria	20 mL/kg of the solution were administered over 6–8 min, at 4–5 °C, through an antegrade route. In case of aortic insufficiency, a retrograde infusion was used	Blood	One liter of the 4:1 (blood:crystalloid) mixture was administered at 4–8 °C. The mixture was delivered through an antegrade route at a pressure of ≤ 300 mmHg. In case of aortic insufficiency, a retrograde infusion was used. A maintenance dose of 200 mL, with lower potassium 25 mEq/500 mL was delivered every 20 min thereafter
Gaudino et al. [19]	Italy	60 patients (31 for HTK, and 29 for MDC)	The study included patients undergoing elective mitral valve surgery. Patients with other valvular, coronary, or carotid pathology, previous cerebrovascular accident, any neurological risk factor, or pre-operative renal insufficiency were excluded	30 ml/Kg of the ice-cold solution were administered over 6–8 min, through an antegrade route, at a pressure of 100–110 mmHg	Blood	An initial 300 mL/min of the warm blood were administered over two minutes through an antegrade route. Subsequent doses of 200 mL/min over two minutes were delivered. Potassium (2 mEq/mL) was added at an initial rate of 150 mL/h, followed by 120, 90, 60 mL/h at subsequent doses, and maintenance rate of 40 mL/h. The doses were delivered every 15 min
Mercan et al. [25]	Turkey	50 patients (25 in each group)	The study included patients undergoing elective CABG, who were aged 40–80 years. Having valvulopathy, LVEF < 30%, chronic renal failure, impaired Liver function test result, emergency or redo surgery, history of cerebrovascular or carotid artery disease, history of cardiopulmonary resuscitation were exclusion criteria	20 ml/kg of the solution were administered through an antegrade route. A terminal warm cardioplegia was delivered	Blood	15 ml/kg of the 4:1 (blood:crystalloid) mixture were administered at 4 °C initially, through an antegrade route. This was followed by a maintenance dose of 10 ml/kg every 20 min. A terminal warm cardioplegia was delivered
Kammerer et al. [20]	Germany	107 patients (55 for HTK, and 52 for MDC)	The study included patients undergoing elective mitral valve surgery. Patients with aortic valvulopathy or coronary artery disease were excluded	Two liters f the solution were administered at 4 °C, through an antegrade route, with a pressure of 50 mmHg	Blood	Warm blood cardioplegia according to the modified Calafiore protocol, at 35 °C. 40 ml of 2 mmol/ml KCl and 10 ml of 2 mmol/ml MgSO4 were delivered. The solution was readministered every 20 min
Braathen et al. [10]	Norway and Sweden	76 patients (38 in each group)	The study included patients undergoing elective mitral valve surgery for mitral regurgitation (≥ grade 3). Patients with other valvulopathy or coronary artery stenosis (50%) were excluded	1800 mL of the solution were administered over 6–8 min, at 4–8 °C, through an antegrade route, with a pressure from approximately 2 m height	Blood	900 mL of the 4:1 (blood:crystalloid) mixture were administered at 4–8 °C, through an antegrade route. A maintenance dose of 500 mL was delivered every 20 min thereafter. The solution was delivered with a pressure of ≤ 300 mmHg
Demmy et al. [21]	US	136 patients (68 in each group)	The study included patients between 35 and 80 years of age, undergoing CABG. Recent MI, valvulopathy that needs surgery, LVEF < 20%, need for mechanical circulatory support, prior CABG surgery, use of aprotinin, participation in another studies within a month of the operation, cardiogenic shock, or severe chronic obstructive lung disease were exclusion criteria	Four liters of the solution were administered over 6–7 min, at 4–6 °C, through an antegrade route, with a pressure < 80 mmHg	Crystalloid	One liter of Plegisol solution was administered at 4–6 °C, through an antegrade route, with a pressure of 80 mmHg. The solution was readministered every 20 min, with a pressure of 40 mmHg. the solution was infused over 1–4 min
Arslan et al. [22]	Turkey	48 patients (21 in each group)	The study included patients undergoing CABG	10–15 mL/kg of the solution were administered as antegrade single doses	Crystalloid	10–15 mL/kg of cold potassium solution were administered through antegrade route
Careaga et al. [23]	Mexico	30 patients (15 in each group)	The study included patients undergoing elective open heart surgery, who had no previous cardiac surgery	300 cc/kg of the solution were administered at 4–8 °C, through an antegrade route, with a pressure of 100 mmHg	Crystalloid	300 cc/m^2^ of cold potassium solution were administered every 20 min, at 4–8 °C, through an antegrade route, with a pressure of 100–120 mmHg
Beyersdorf et al. [24]	Germany	37 patients (12 for HTK, and 12 for MDC)	The study included patients undergoing CABG	Two liters of the solution were administered at 4–6 °C, with a pressure of 100 mmHg initially, and 50 mmHg after cardiac asystole	Blood	250–300 mL/min of the 4:1 (blood:crystalloid) mixture were administered initially over 3 min, at 8–14 °C, with a pressure of 100 mmHg. Thereafter, a repeated dose of 200 mL/min over 2 min was delivered every 20 min. Following asystole, the potassium dose was reduced
Gallandat huet et al. [6]	Netherlands	249 patients (132 for HTK, and 117 for MDC)	The study included patients undergoing CABG	20–25 mL/kg of the solution were administered at 4 °C, through an antegrade route, by gravity from a height of 1.5 m. Reinfusion dose of 300–500 mL was delivered after 45 min or when needed. The total final amount is about 2500 ml	Crystalloid	One liter of ST.Thomas solution (alkalinized with 10 mmol/I sodium bicarbonate) was administered at 4 °C, through an antegrade route, with a pressure of 150 mmHg. The solution infused with a pressure bag at 150 mm Hg. The pressure in the aorta root is then about 70 mmHg (13). Reinfusion dose of 300–500 mL was delivered after 45 min or when needed. The total final amount is about 1700 ml

HTK solution: Histidine–tryptophan–ketoglutarate solution, MDC: multiple dose cardioplegia, CABG: coronary artery bypass grafting, LV: left ventricle, LVEF: left ventricular ejection fraction, MI: myocardial infarction, US: United States

**Table 2:** shows baseline characteristics of enrolled patients

Study ID	Study arms	Age (years)	Male sex	Weight (Kg)	Medical history				NYHA class				
					Smoking	Diabetes mellitus	Hypertension	Hyperlipidemia	1	2	3	4	Average
Ali et al. [16]	HTK	44.19 ± 11.63	118 (73.8%)	–	56 (35%)	72 (45%)	80 (50%)	–	8 (5%)	56 (35%)	80 (50%)	16 (10%)	–
	MDC	43.11 ± 10.7	120 (75%)	–	40 (25%)	56 (35%)	72 (45%)	–	8 (5%)	80 (50%)	56 (35%)	16 (10%)	–
Cvetković et al. [17]	HTK	64.5 ± 6.5	40 (74.1%)	–	21 (38.9%)	24 (44.4%)	40 (74.1%)	24 (44.4%)	–	–	–	–	1.57 ± 0.6
	MDC	65.3 ± 6.3	44 (88%)	–	21 (42%)	20(40%)	44 (88%)	24 (48%)	–	–	–	–	1.6 ± 0.62
Vivacqua et al. [18]	HTK	63 ± 13	29 (52.7%)	83 ± 17	–	11 (20%)	32 (58.2%)	39 (70.9%)	–	–	–	–	–
	MDC	70 ± 11	35 (63.6%)	90 ± 22	–	12 (21.8%)	45 (81.8%)	44 (81.5%)	–	–	–	–	–
Gaudino et al. [19]	HTK	64 ± 9	25 (80.7%)	–	–	–	–	–	–	–	15 (48.4%)	9 (29%)	–
	MDC	61 ± 5	21 (72.4%)	–	–	–	–	–	–	–	18 (62.1%)	7 (24.1%)	–
Mercan et al. [25]	HTK	60.1 ± 7.8	19 (76%)	–	–	12 (48%)*	17 (68%)	9 (36%)	–	–	–	–	–
	MDC	62.7 ± 9.4	21 (84%)	–	–	13 (52%)*	12 (48%)	12 (48%)	–	–	–	–	–
Kammerer et al. [20]	HTK	65 ± 14	31 (56.4%)	74 ± 13	–	5 (9.1%)	–	–	–	–	–	–	–
	MDC	66 ± 9	36 (69.2%)	77 ± 19	–	4 (7.7%)	–	–	–	–	–	–	–
Braathen et al. [10]	HTK	59 ± 2	34 (89.5%)	86 ± 3	–	–	–	–	–	–	–	–	–
	MDC	59 ± 2	25 (65.8%)	80 ± 2	–	–	–	–	–	–	–	–	–
Demmy et al. [21]	HTK	62	67 (98.5%)	–	–	–	–	–	–	–	–	–	–
	MDC		61 (89.7%)	–	–	–	–	–	–	–	–	–	–
Arslan et al. [22]	HTK	60.23 ± 5.6	16 (76.2%)	78.4 ± 11.9	–	–	–	–	–	–	–	–	–
	MDC	60.38 ± 7.3	19 (90.5%)	75.6 ± 13.2	–	–	–	–	–	–	–	–	–
Careaga et al. [23]	HTK	53 ± 19.75	21 (70%)	–	–	–	–	–	–	–	–	–	–
	MDC			–	–	–	–	–	–	–	–	–	–
Beyersdorf et al. [24]	HTK	58 ± 7	9 (75%)	–	7 (58.3%)	0 (0%)	3 (25%)	7 (58.3%)	0 (0%)	0 (0%)	12 (100%)	0 (0%)	–
	MDC	59 ± 8	9 (75%)	–	8 (66.7%)	2 (16.7%)	4 (33.3%)	10 (83.3%)	0 (0%)	4 (33.3%)	7 (58.3%)	1 (8.3%)	–
Gallandat huet et al. [6]	HTK	60.7 ± 8.8	107 (81.1%)	–	–	–	–	–	–	–	–	–	–
	MDC	60.7 ± 7.6	94 (80.3%)	–	–	–	–	–	–	–	–	–	–

Data were presented as mean ± standard deviation or number (%)

NYHA: New York Heart Association Functional Classification

*Type-2 diabetes mellitus

### Quality assessment (risk of bias)

**Fig. 2:**
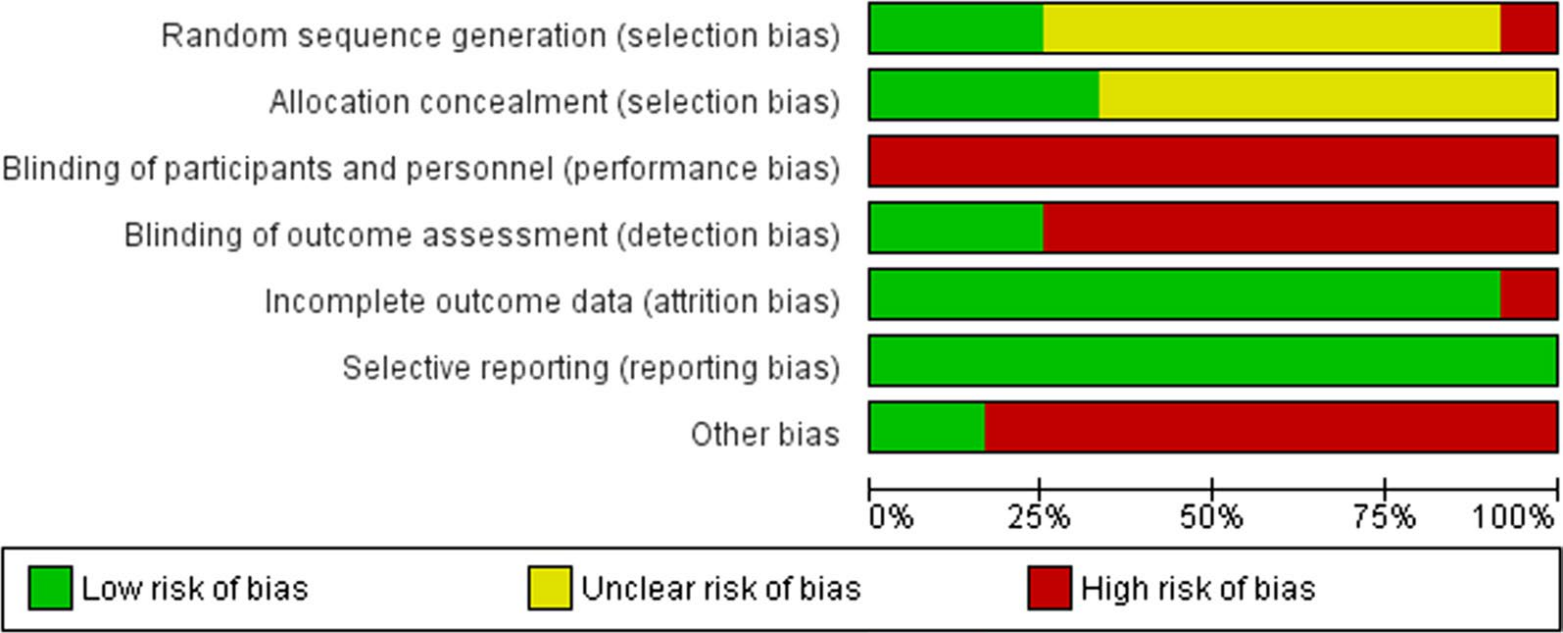
Risk of bias graph: review authors' judgments about each risk of bias item presented as percentages across all included studies

**Fig. 3:**
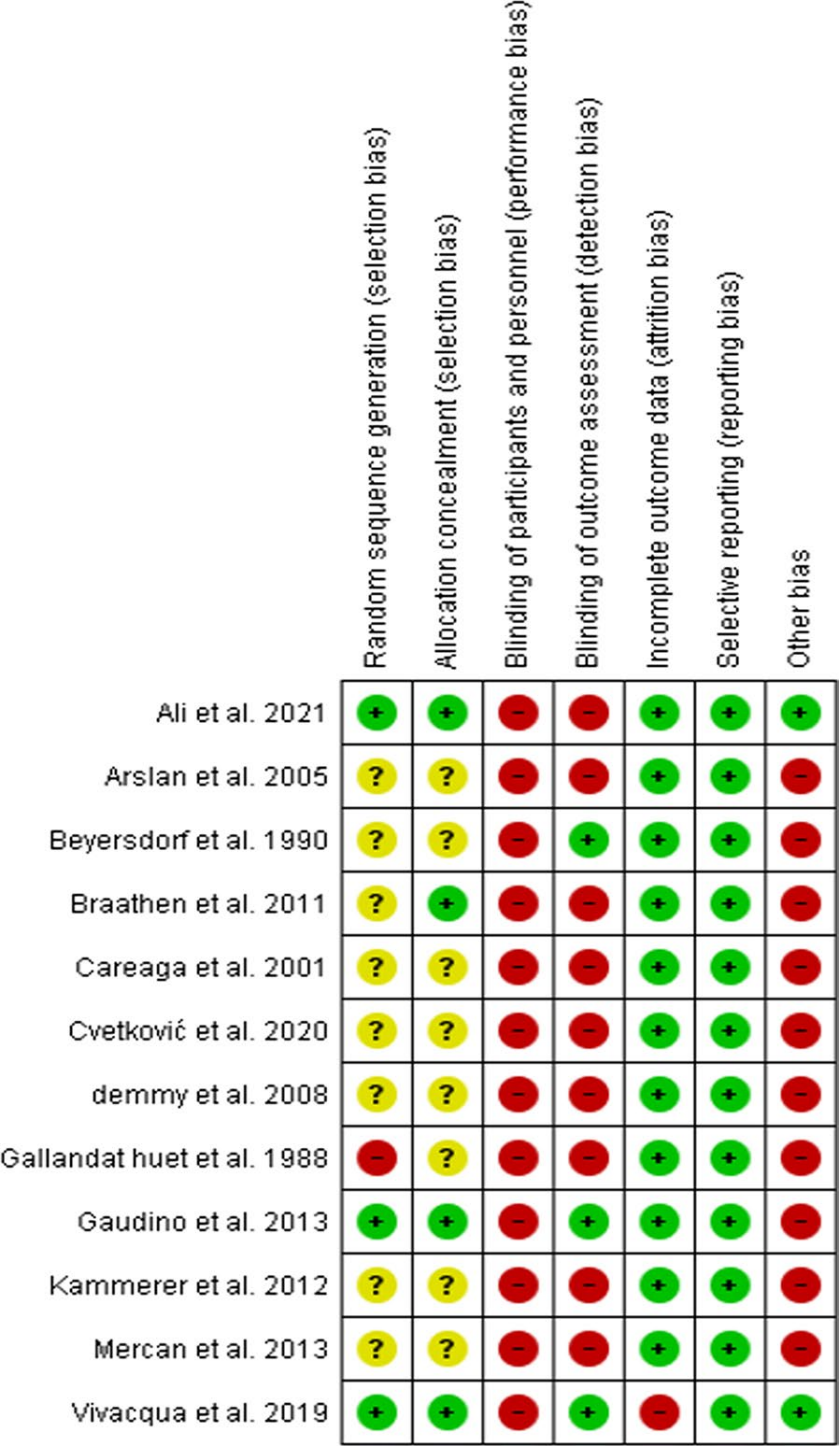
Risk of bias summary: review authors' judgements about each risk of bias item for each included study

Generally, the included trials had a low risk of reporting and attrition bias, and a low to moderate risk of selection bias. However, a potential source of performance bias was the inability to blind the participants and personnel. In most of the studies, the lack of blinding the outcomes assessors might have induced some detection bias. Having no registered protocol available was a potential source of bias as well in most of the studies. The risk of bias graph and summary are shown in Figs. 2, and 3 respectively.

### Study outcomes

#### CPB time (min)

**Fig. 4:**
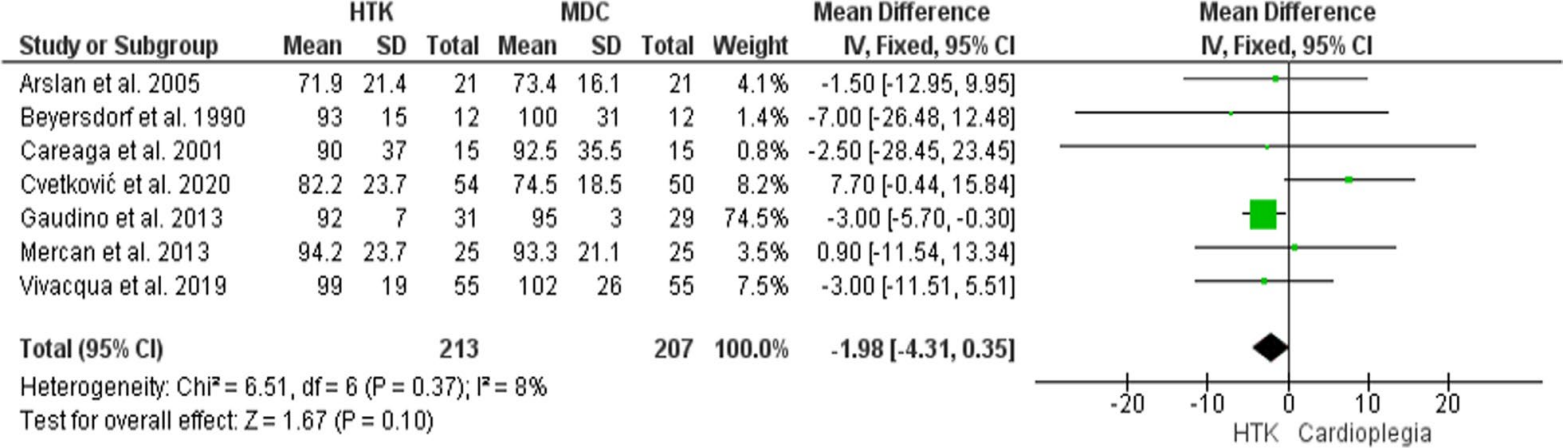
Forest plot of the comparison: HTK versus MDC, outcome: CPB time (min)

Seven trials were included in this analysis, with 420 patients enrolled (213 for HTK, and 207 for MDC). The two interventions did not differ significantly in CPB time (MD = − 1.98; 95% CI [− 4.31, 0.35], p = 0.1), and the result were homogenous (P = 0.37, I^2^ = 8%) (Fig. 4).

#### Aortic cross-clamping time (min)

**Fig. 5:**
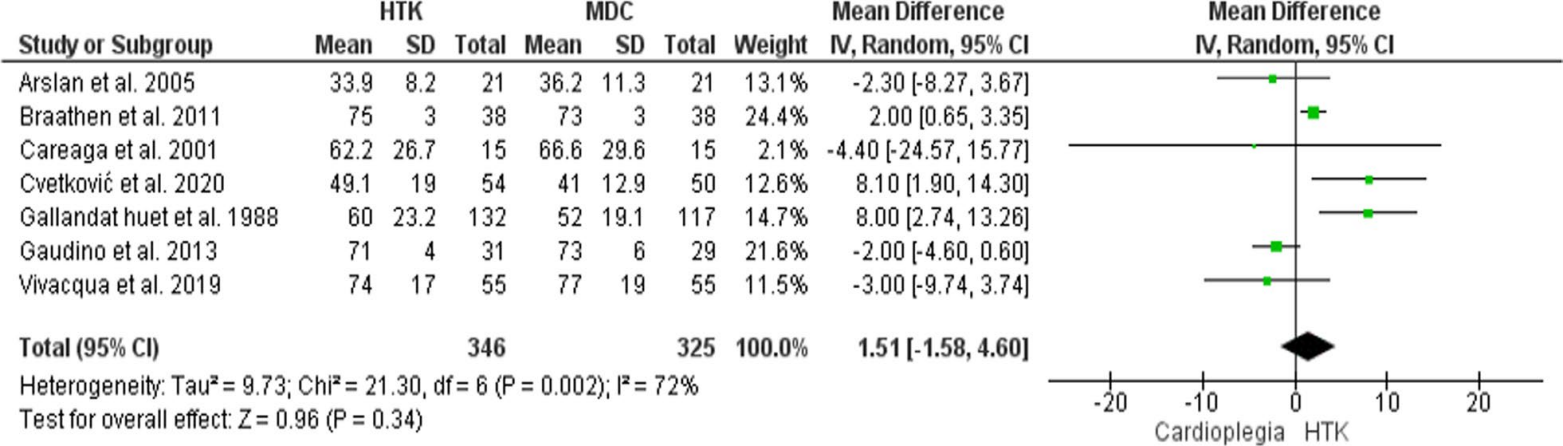
Forest plot of the comparison: HTK versus MDC, outcome: Aortic cross-clamping time (min)

The analysis of this outcome included seven trials, with 671 patients enrolled (346 for HTK, and 325 for MDC). The comparative meta-analysis revealed no significant difference in Aortic cross-clamping time between the two interventions (MD = 1.51; 95% CI [− 1.58, 4.60], p = 0.34). However, the results were heterogeneous across the trials (P = 0.002, I^2^ = 72%) (Fig. 5).

#### Cardiac arrest beginning time (s):

**Fig. 6:**
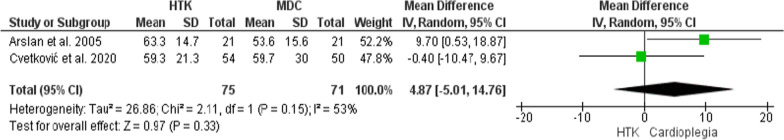
Forest plot of the comparison: HTK versus MDC, outcome: Cardiac arrest beginning time (s)

Two trials participated with analyzable data in this outcome, with 146 patients enrolled (75 for HTK, and 71 for MDC). The analysis showed no significant difference between the two interventions in cardiac arrest beginning time (MD = 4.87; 95% CI [− 5.01, 14.76], p = 0.33). There was significant heterogeneity across the trials (P = 0.15, I^2^ = 53%) (Fig. 6).

#### Number of grafts

**Fig. 7:**
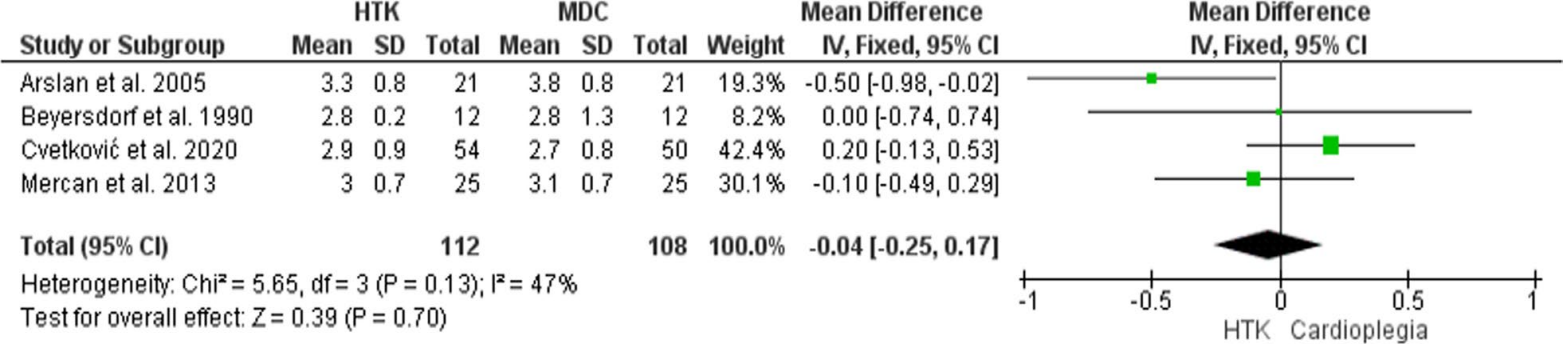
Forest plot of the comparison: HTK versus MDC, outcome: Number of grafts

The analysis of this outcome was conducted upon four trials, with 220 patients enrolled (112 for HTK, and 108 for MDC). The meta-analysis showed no significant difference in the number of grafts between the two interventions (MD = − 0.04; 95% CI [− 0.25, 0.17], p = 0.7), and the results were homogenous (P = 0.13, I^2^ = 47%) (Fig. 7).

#### Postoperative inotropic support

**Fig. 8:**
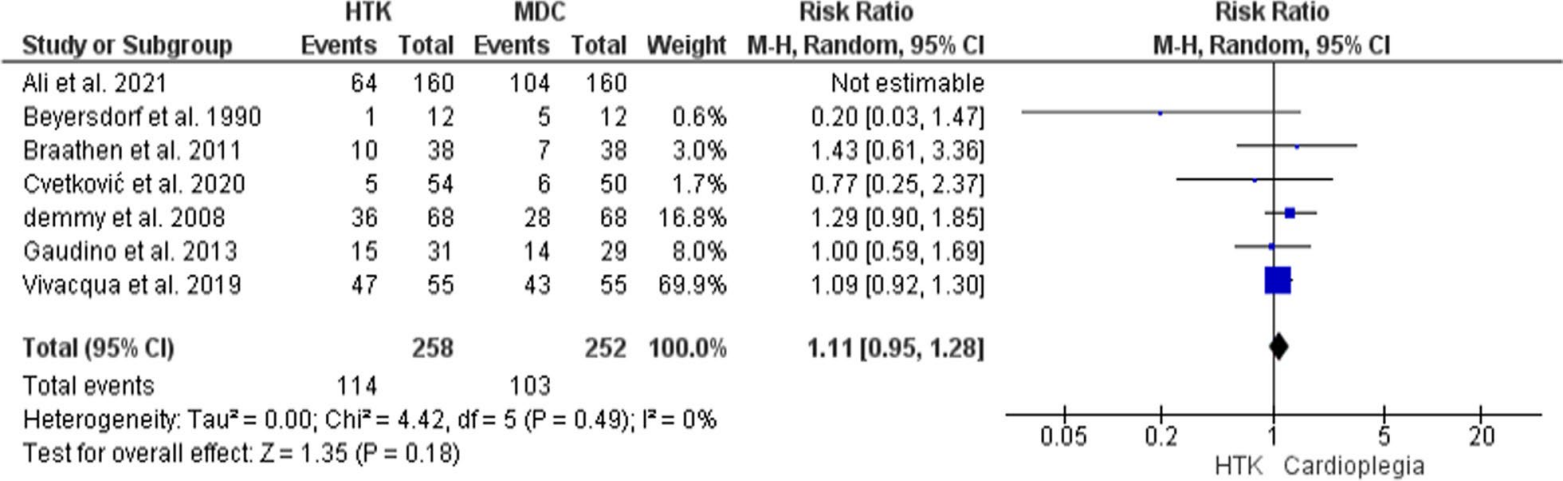
Forest plot of the comparison: HTK versus MDC, outcome: postoperative inotropic support

The primary analysis of this outcome included seven trials, with 830 patients enrolled (418 for HTK, and 412 for MDC). The two interventions did not vary significantly in the risk for postoperative inotropic support (RR = 0.94; 95% CI [0.67, 1.31], p = 0.71), but the results were heterogeneous (P = 0.0004, I2 = 76%). A sensitivity analysis was conducted by excluding Ali et al. 2021 [16], which resolved the heterogeneity without affecting the significance of the pooled estimate (RR = 1.11; 95% CI [0.95, 1.28], p = 0.18), (P = 0.49, I^2^ = 0%) (Fig. 8).

#### EF change (%)

**Fig. 9:**
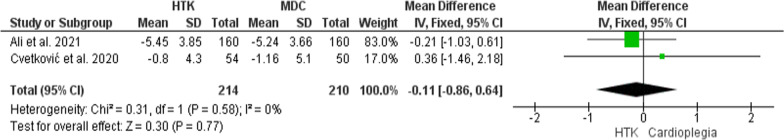
Forest plot of the comparison: HTK versus MDC, outcome: EF change (%)

Two trials were included in the analysis of this outcome, with 146 patients enrolled (75 for HTK, and 71 for MDC). The analysis showed no significant difference in EF change between the two interventions (MD = − 0.11; 95% CI [− 0.86, 0.64], p = 0.77), and the results were homogenous (P = 0.58, I^2^ = 0%) (Fig. 9).

#### ECG change

**Fig. 10:**
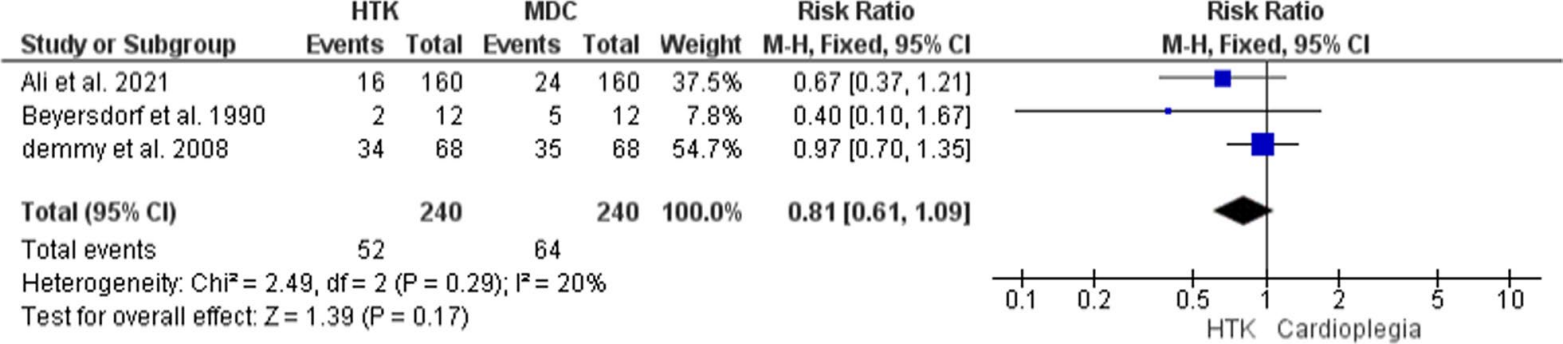
Forest plot of the comparison: HTK versus MDC, outcome: ECG change

Three trials contributed with analyzable data to this analysis, with 480 patients enrolled (240 in each group). In terms of effectiveness, there was no significant difference between the two methods in ECG changes (RR = 0.81; 95% CI [0.61, 1.09], p = 0.17), and the results were homogenous (P = 0.29, I^2^ = 20%) (Fig. 10).

#### Postsurgical atrial fibrillation

**Fig. 11:**
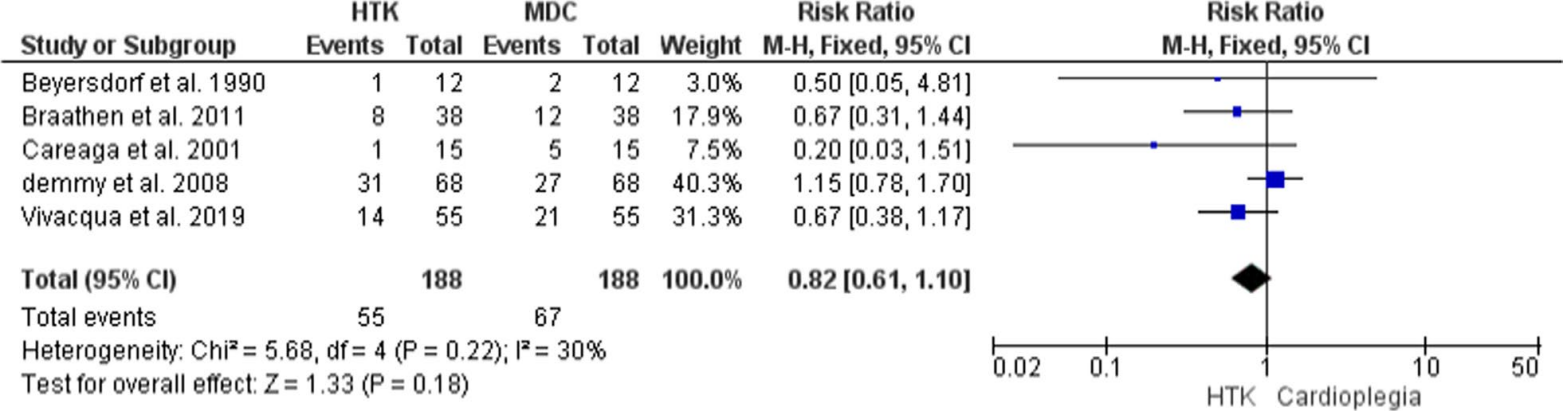
Forest plot of the comparison: HTK versus MDC, outcome: Postsurgical atrial fibrillation

The analysis of this outcome was based upon five trials, with 376 patients enrolled (188 in each group). The comparative meta-analysis revealed no significant difference between the two interventions in the risk of postsurgical atrial fibrillation (RR = 0.82; 95% CI [0.61, 1.10], p = 0.18), and the results were homogenous (P = 0.22, I^2^ = 30%) (Fig. 11).

#### Hospital stay (days)

**Fig. 12:**
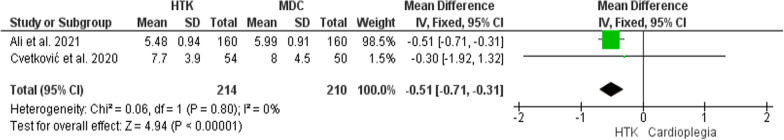
Forest plot of the comparison: HTK versus MDC, outcome: Hospital stay (days)

Two studies were involved in the analysis of hospital stay days, with 424 patients enrolled (214 for HTK, and 210 for MDC). HTK solution administration has resulted significantly in shorter hospital stay (MD = − 0.51; 95% CI [− 0.71, − 0.31], p < 0.00001), and the results were highly homogenous (P = 0.8, I^2^ = 0%) (Fig. 12).

#### ICU stay (days)

**Fig. 13:**
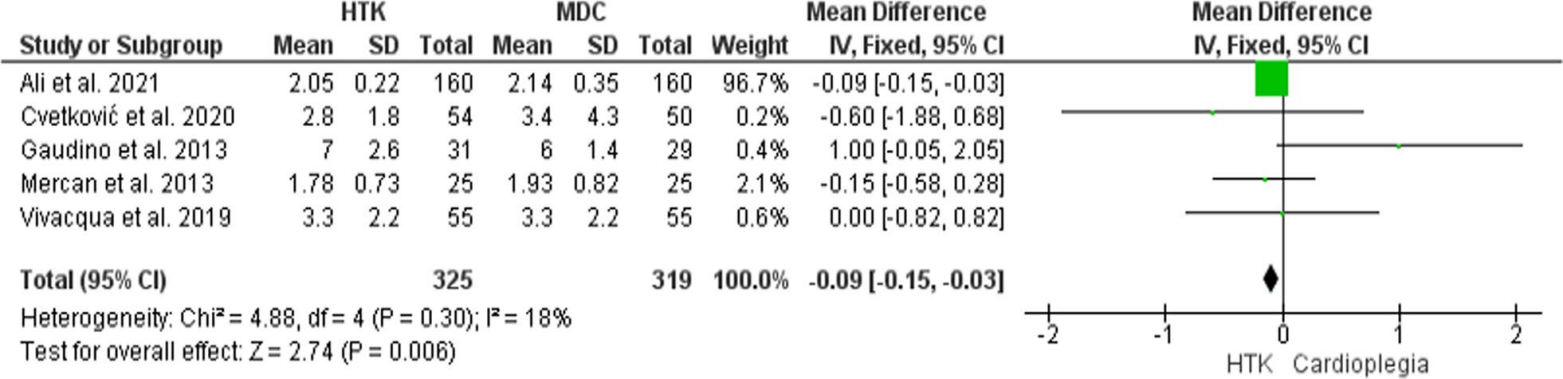
Forest plot of the comparison: HTK versus MDC, outcome: ICU stay (days)

This analysis was conducted upon five trials, with 466 patients enrolled (325 for HTK, and 319 for MDC). HTK solution has significantly resulted in shorter ICU stay (MD = − 0.09; 95% CI [− 0.15, − 0.03], p = 0.006), and the results were homogenous (P = 0.3, I^2^ = 18%) (Fig. 13).

#### CK level (IU/L)

The analysis of this outcome was based upon three trials, with 173 patients enrolled (88 for HTK, and 85 for MDC).

**After 4–7 h:** HTK solution has resulted significantly in lower level of CK (MD = − 157.52; 95% CI [− 272.31, − 42.19], p = 0.007), but the results were heterogeneous (P = 0.003, I^2^ = 82%).

**After 24 h:** Initially, the two interventions did not differ significantly in the release of CK (MD = -14.79; 95% CI [− 345.14, 315.56], p = 0.93), but the results were heterogeneous (P = 0.002, I2 = 83%). Thereafter, Beyersdorf et al. 1990 [24] was excluded in a sensitivity analysis, in which the results were homogenous in favor of HTK solution (MD = − 136.62; 95% CI [− 267.20, − 6.05], p = 0.04), (P = 0.44, I2 = 0%).

**Fig. 14:**
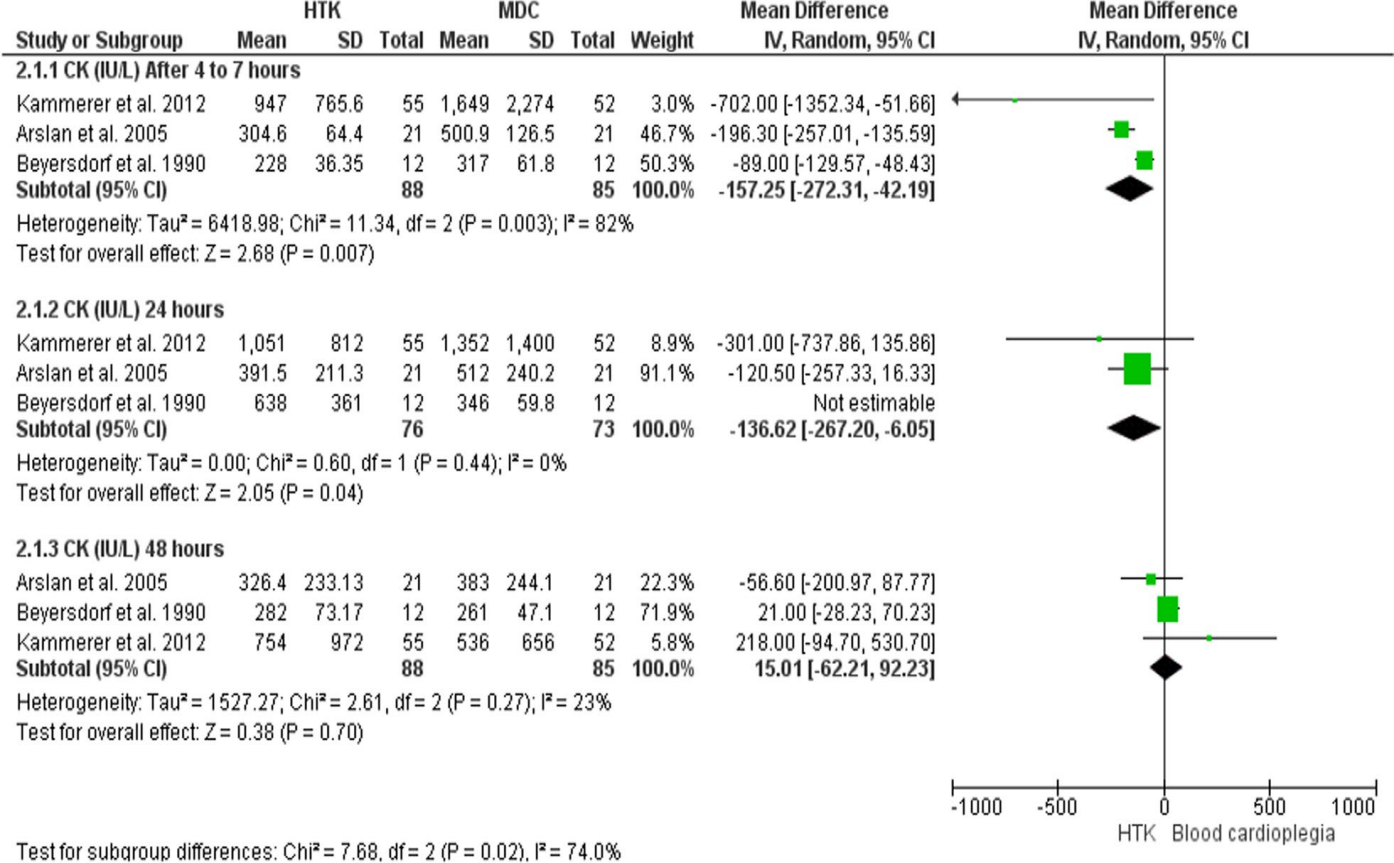
Forest plot of the comparison: HTK versus MDC, outcome: CK level (IU/L)

**After 48 h:** The meta-analysis showed no meaningful difference in CK release between the two interventions (MD = 15.01; 95% CI [− 62.21, − 92.23], p = 0.7), and the results were homogenous (P = 0.27, I^2^ = 23%) (Fig. 14).

#### CK-MB level (ng/ml)

Three trials were included in this analysis, with 204 patients enrolled (100 for HTK, and 104 for MDC).

**After 4–8 h:** The primary analysis of this outcome showed no significant difference in CK-MB level between the two interventions (MD = − 6.82; 95% CI [− 14.69, 1.05], p = 0.09), but the results were heterogeneous (P = 0.04, I^2^ = 69%). Beyersdorf et al. 1990 [24] was excluded in a sensitivity analysis, which resolved the heterogeneity without changing the significance of the pooled estimate (MD = − 2.41; 95% CI [− 9.08, 4.27], p = 0.48), (P = 0.93, I^2^ = 0%).

**After 20–24 h:** The two interventions did not differ significantly in releasing CK-MB (MD = 3.29; 95% CI [− 0.56, 7.14], p = 0.09), and the results were homogenous (P = 0.81, I^2^ = 0%).

**Fig. 15:**
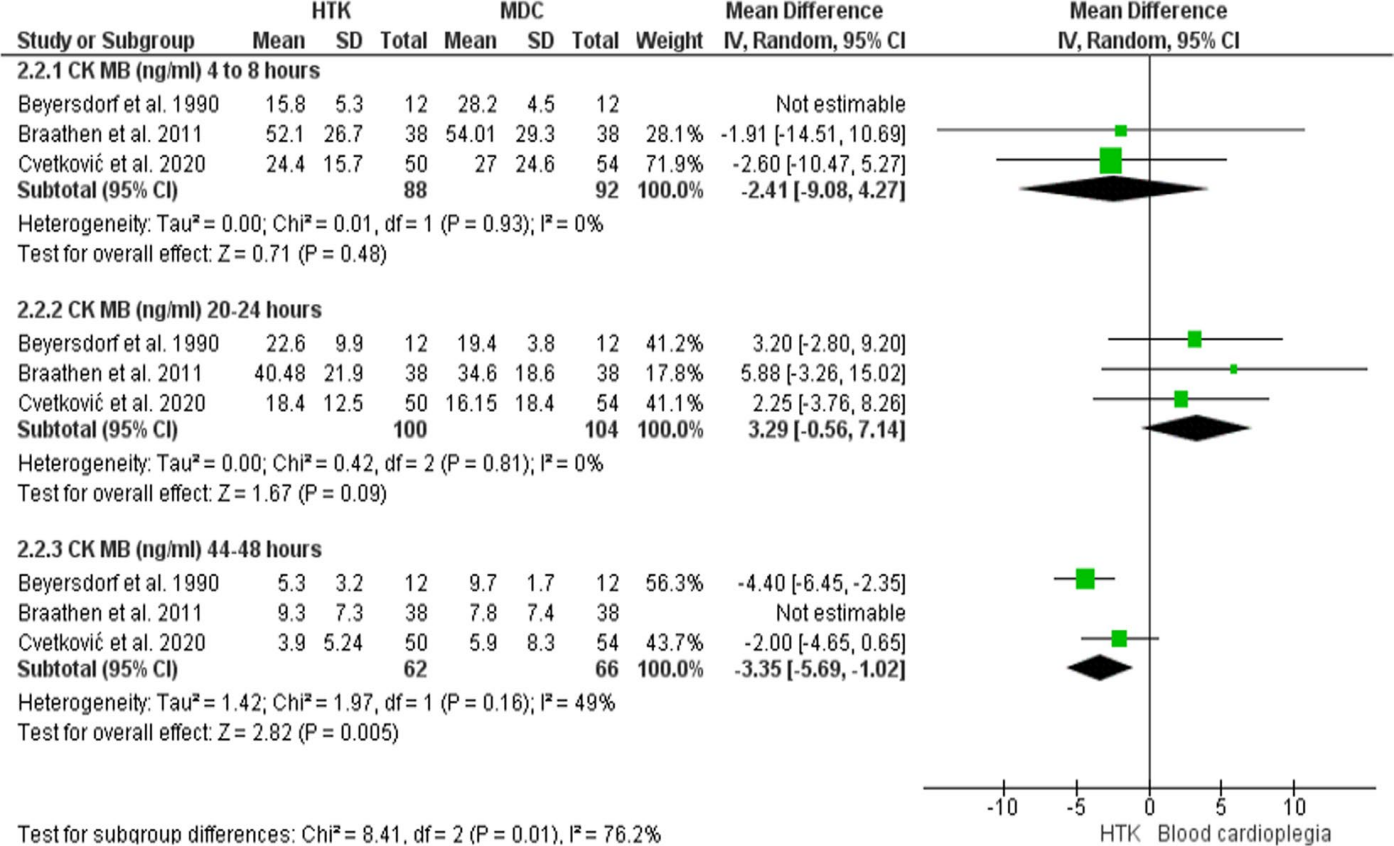
Forest plot of the comparison: HTK versus MDC, outcome: CK-MB level (ng/ml)

**After 44–48 h:** The analysis revealed no significant difference between the two interventions in releasing CK-MB (MD = − 1.84; 95% CI [− 5.08, 1.39], p = 0.26), but the results were heterogeneous (P = 0.01, I2 = 78%). A sensitivity analysis was conducted by excluding Braathen et al. [10], in which the heterogeneity was resolved in favor of HTK solution (MD = − 3.35; 95% CI [− 5.69, − 1.02], p = 0.005), (P = 0.16, I^2^ = 49%) (Fig. 15).

#### Tn-I level (ng/ml)

Three trials had participated with analyzable data for this outcome, with 282 patients enrolled (139 for HTK, and 143 for MDC).

**After 4–7 h** The release of Tn-I did not differ considerably between the two interventions (MD = 0.25; 95% CI [− 1.92, 2.42], p = 0.82), but the results were heterogeneous (P = 0.03, I^2^ = 71%). A sensitivity analysis was conducted by excluding Arslan et al. 2005 [22], in which the heterogeneity was resolved and the significance of the pooled estimate remained unchanged (MD = − 0.73; 95% CI [− 1.69, 0.23], p = 0.14), (P = 0.86, I^2^ = 0%).

**After 24 h:** The comparative analysis showed no significant difference between the two interventions in releasing Tn-I (MD = − 0.36; 95% CI [− 1.48, 0.76], p = 0.53), and the results were homogenous (P = 0.98, I^2^ = 0%).

**Fig. 16:**
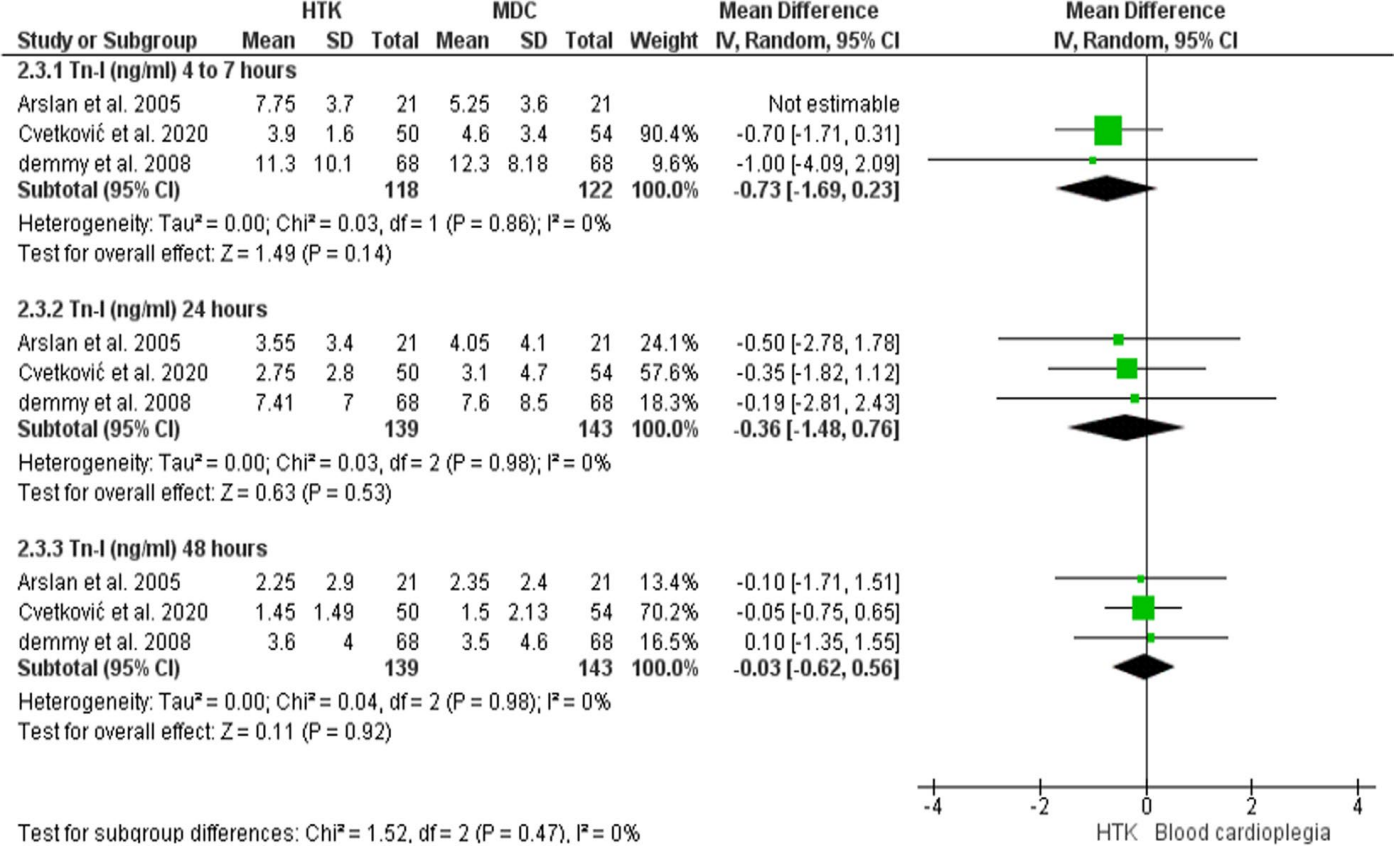
Forest plot of the comparison: HTK versus MDC, outcome: Tn-I level (ng/ml)

**After 48 h:** the two interventions did not vary significantly in releasing Tn-I (MD = − 0.03; 95% CI [− 0.62, 0.56], p = 0.92), and the results were homogenous (P = 0.98, I^2^ = 0%) (Fig. 16).

## Discussion

This systematic review and meta-analysis provides an update to the current evidence by summarizing the findings of 12 RCTs that compared HTK solution to other cardioplegic solutions in various cardia surgeries. Data from 1,327 cardiac patients were summarized, among them, 666 patients had received the HTK solution. HTK solution has resulted significantly in shorter hospital (p < 0.00001) and ICU (p = 0.006) stay. Moreover, in comparison with other cardioplegic solutions, the HTK solution has significantly decreased the release of CK (after 4–7 h (p = 0.007), and 24 h (p = 0.04)), as well as CK-MB (after 44–48 h (p = 0.005)). These findings indicate superiority in myocardial protection at the biochemical level.

This article updates the previous meta-analysis Reynolds et al. 2020 (26), with four added RCTs [16, 17, 23, 25]. Our findings were consistent with the previous ones to a large extent. However, our update revealed the significant role of the HTK solution in reducing the release of CK-MB; an outcome that was insignificant in the previous meta-analysis. Furthermore, this updated review has investigated more outcomes than those reported previously. Among these outcomes were the hospital and ICU stay duration, which favored the HTK solution. Other newly investigated outcomes were cardiac arrest beginning time, number of grafts, EF change, and ECG change.

HTK solution was found to be as effective as other in-use-cardioplegic solutions. Moreover, it provides longer protection for the myocardium. This long protection makes it easier to administer, with minimal interruption of the surgical site. Furthermore, the analysis showed a superiority of the solution in shortening the recovery period, given the shorter ICU stay duration. Hospital stay days were reduced as well, which prevents the acquisition of nosocomial infection, and deterioration of physical and psychological health. Added to that, HTK solution protects the heart more, given the lower level of cardiac enzymes detected in the serum.

Among the included studies, Huet et al. [6] and Cvetković et al. [17] have compared HTK with St Thomas cardioplegia and concluded that no difference between both solutions in terms of safety, efficacy, and hemodynamics.

This review was strengthened by including only experimental controlled trials with adequate randomization. The selected studies design provides the highest power of evidence. However, this review was limited by the variation between the included trials data in some outcomes. This heterogeneity could not be resolved on some occasions. The inability to blind the study participants and personnel, as well as the outcome assessors in the majority of the trials was a probable source of bias. Most of the trials had no registered protocol. Due to the difference between cardioplegic solutions compared to HTK, a subgroup analysis could not performed according to the comparator. Therefore, further studies are recommended to compare HTK to the most frequently used solutions, such as St Thomas and BuckBerg, in order to determine the best option for each case and surgery.

The study concluded that, HTK solution had the same efficacy and safety as the in-use-cardioplegic solutions in most of the measured parameters. Furthermore, HTK solution showed superiority in reducing ICU and hospital stay, as well as CK and CK-MB release. Given its high efficacy and simple administration, the HTK solution constitutes an important alternative for MDC.

## Data Availability

Not applicable.
